# Current advances of photobiomodulation therapy in treating knee osteoarthritis

**DOI:** 10.3389/fcell.2023.1286025

**Published:** 2023-11-16

**Authors:** Yanlei Zhang, Quanbo Ji

**Affiliations:** ^1^ Medical School of Chinese PLA, Beijing, China; ^2^ Department of Orthopedics, The General Hospital of Chinese PLA, Beijing, China

**Keywords:** knee osteoarthritis, photobiomodulation therapy, synovial inflammation, pain, macrophage polarization

## Abstract

Knee osteoarthritis (KOA) is manifested by low-grade joint inflammation, irreversible cartilage degeneration, subchondral bone remodeling and osteophyte formation. It is one of the most prevalent degenerative diseases in the elderly. KOA usually results in chronic joint pain, physical impairment even disability bringing a huge socioeconomic burden. Unfortunately, there is so far no effective interventions to delay the progression and development of KOA. There is a pressing need for explorations and developments of new effective interventions. Photobiomodulation therapy (PBMT), also known as low-level light therapy (LLLT), has attracted widespread attention in treating KOA because it is drug-free, non-invasive, safe and useful with rarely reported side effects. It provides the biological stimulatory effects primarily by enhancing the activity of mitochondrial cytochrome c oxidase. This stimulation, in turn, fosters cell proliferation and tissue regeneration. In addition to this, the paper provides a concise overview of the light parameters and the effectiveness of PBMT when applied in the treatment of KOA patients in clinical settings. It also delves into the experimental evidence supporting the modulatory effects of PBMT and its potential underlying mechanisms in addressing synovitis, cartilage degeneration, and pain resolution.

## 1 Introduction

Osteoarthritis (OA) is an age-related degenerative disease and the primary cause of pain and physical impairment in the elderly (Fang and Beier, 2014). Knee osteoarthritis (KOA) is the most common type of OA and accounts for 85% of the incidences of OA, affecting 37% of the people over sixty (GBD, 2015 Disease and Injury Incidence and Prevalence Collaborators, 2016; Sharma, 2021). According to an epidemiological study, the overall prevalence of KOA in Chinese people over forty-five was 8.1% (Tang et al., 2016), which will definitely lead to a huge social and economic burden. KOA is mainly characterized by pathologic changes including synovitis, cartilage degeneration, subchondral bone remodeling and osteophyte formation, leading to joint pain, swelling, stiffness, physical impairments, loss of quality of life and even disability (Katz et al., 2021). Unfortunately, to date, there’s no disease-modified anti-KOA drugs for treating KOA patients. Current therapeutics for KOA, including conservative non-pharmacy therapy (for instance exercise), medication and surgery, usually focus on relieving pain and improving quality of life. Non-steroid anti-inflammatory drugs (NSAIDs) are recommended in most of the clinical guidelines for KOA, however, it’s accompanied with unavoidable potential risks of side effects including gastrointestinal bleeding (da Costa et al., 2021; Khumaidi et al., 2022). Surgery, total knee replacement (TKR) in particular, was thought to be the most effective intervention for advanced or end-stage KOA patients, but 10%–34% patients after TKR reported an unfavorable long-term pain outcome (Beswick et al., 2012). Therefore, research and development of new interventions are urgently required.

Photobiomodulation therapy (PBMT), also known as low-level light therapy (LLLT), is a kind of physical therapy applying red or near-infrared (NIR) light from laser or Light Emitting Diode (LED) (Cardoso et al., 2021). PBMT has been widely applied in treating diabetic ulcer (Peplow et al., 2012), neck pain (Chow et al., 2009), age-related neurocognitive impairments (Cardoso et al., 2021), OA (Stelian et al., 1992) and so on, exerting eye-catching effects in clinical application due to its non-invasive, painless and drug-free properties with rarely reported serious events (Chow et al., 2009; Hamblin, 2013; DE Oliveira et al., 2022). In contrast with high-intensity light therapy (HILT), PBMT is shown to penetrate the superficial tissue and to meanwhile activate the biostimulatory, anti-inflammatory and regenerative responses without generating any heating sensation (Ahmad et al., 2022; Ahmad et al., 2023; Zhang and Qu, 2023). The mechanisms of PBMT are not totally uncovered so far. Ample experimental evidence suggests that cytochrome c oxidase (CCO), the key enzyme in mitochondrial respiratory chain, acts as the acceptor of photons (Karu, 1999; Hanna et al., 2021). And the light absorption later results in increased production of adenosine triphosphate (ATP), release of nitric oxide (NO), induction of transcription factors and modulation of reactive oxygen species (ROS) (Karu, 2008; Kim et al., 2017). Apart from this, PBMT also displays its therapeutic effects through regulating angiogenesis, modulating blood flow and stimulating the expression of inflammatory factors (Zhang and Qu, 2023).

During the past 30 years, cumulative researches have investigated the effects of PBMT in treating KOA in both patients and experimental animals, indicating the cardinal roles of PBMT in attenuating KOA inflammation and pain (Hamblin, 2013; Zhang and Qu, 2023). However, it’s still controversial when it comes to the optimal dosage. In addition, the potential mechanisms of PBMT in KOA are also under explorations. In this narrative review, light parameters and effectiveness of PBMT in treating KOA patients are summarized and discussed. Experimental evidence of the biomodulatory effects and potential underlying mechanisms of PBMT in attenuating pain, synovitis and cartilage regeneration are also briefly introduced.

## 2 Light parameters and effectiveness of PBMT in treating KOA patients

A series of randomized controlled trials (RCTs) are conducted in the past three decades to evaluate the effectiveness of PBMT in treating KOA patients. The light parameters used in these researches are summarized in Table 1. In 1992, Stelian and colleagues performed the first RCT of PBMT for treating KOA (Stelian et al., 1992). They recruited fifty KOA patients and gave them 633 nm He-Ne laser, 830 nm Ga-Al-As laser, placebo laser treatment respectively twice a day for 15 min per time. After the 10-day treatments, pain was remarkably reduced by more than fifty percent and physical functions of patients were significantly improved in both laser therapy groups. These results suggest that PBMT is effective in relieving KOA pain and improving physical functions. In a descriptive, prospective study, eighteen KOA patients were treated with PBMT using a 810 nm and 890 nm Ga-Al-As diode laser device (Soleimanpour et al., 2014). The patients received PBMT three times per week for a total of twelve sessions. At the end of the treatments, a significant reduction of nocturnal pain, pain on walking and ascending the steps, knee circumference, distance between the hip and heel, and knee to horizontal hip to heel distance was demonstrated, indicating the effectiveness of PBMT. Results of several meta-analysis articles also suggest PBMT is sufficient to remarkably ameliorate joint pain in KOA patients, largely improve Western Ontario and McMaster Universities Arthritis Index (WOMAC) stiffness and functional scores (Rayegani et al., 2017; Stausholm et al., 2019). However, the discrepancy of efficacy still exists. In a randomized, double-blind placebo-controlled study by Bülow et al. (1994), no significant differences in any of the effect variables including pain and joint mobility were found between the PBMT group and control group during or after the treatment. In terms of the overall assessment of the patients, both groups showed a clearly demonstrable positive effect, which might be a result of the placebo effect (Bülow et al., 1994). In Tascioglu’s study (Tascioglu et al., 2004), treating KOA patients five times per week with ten treatments in total using an 830 nm Gal-Al-As diode laser device did not significantly improve the clinical assessments including Visual Analogue Scale (VAS) pain, WOMAC pain, stiffness and physical function subscales, suggesting that PBMT with this chosen laser type and dose regimen had no effects on pain in KOA patients. Some other studies also showed that the combination of PBMT did not optimize the positive therapeutic effects of single exercise therapy for KOA (Vassão et al., 2020a; Vassão et al., 2020b; Malik et al., 2023). In a systematic review by Huang et al. (2015), they analyzed nine RCTs and found no evidence to support the effectiveness of PBMT in improving patients’ WOMAC pain, stiffness, and functional scores. There was also no significant difference in the VAS pain scores between the PBMT and control group 12 weeks after treatment. Therefore, they concluded that the current available evidence did not support the effectiveness of PBMT for KOA patients.

**TABLE 1:** Summary of the light parameters used in RCTs applying PBMT to treat KOA patients.

Study	Sample size	Light source	Wavelength (nm)	Output power (mW)	Spot area (cm^2^)	Power density (mW/cm^2^)	Energy density (J/cm^2^)	Total energy per point (J)	Number of points	Duration-frequency-sessions	Major results
Stelian et al. (1992)	50	Laser	633	18/75	—	8/34	5.1	—	—	15 min-2/d-20	Pain reduction in both laser groups by more than 50%. Significant functional improvement
		Laser	830	25/270	—	11/122	5.6	—	—		
Bülow et al. (1994)	29	Laser	—	—	—	—	—	—	—	15 min-3/w-9	The positive effect of treatment is likely to be due to a placebo effect
Gur et al. (2003)	90	Laser	904	10	1	—	—	—	2	5 min-5/w-10	Significant improvements in pain, function, and quality of life
		Laser	904	11.2	1	—	—	—	2	3 min-5/w-10	
Tascioglu et al. (2004)	60	Laser	830	50	Diameter = 1 mm	—	—	3	5	2 min/point-5/w-10	No significant improvement is observed
		Laser	830	50	Diameter = 1 mm	—	—	1.5	5	1 min/point-5/w-10	
Yurtkuran et al. (2007)	55	Laser	904	4	—	10	1.2	0.48	10	2 min/point-5/w-10	Effective only in reducing periarticular swelling
Fukuda et al. (2011)	47	Laser	904	60	0.5	120	6	3	9	50 s/point-3/w-9	PBMT significantly improves pain and function over the short term
Alfredo et al. (2012)	40	Laser	904	60	0.5	—	6	3	9	50 s/point-3/w-9	PBMT associated with exercises is effective in yielding pain relief, function and activity
Gworys et al. (2012)	94	Laser	810	400	0.63	634.9	12.7	8	12	20 s/point-5/w-10	Significant improvements in knee joint function and pain relief
			808 + 905	1,100	2	—	6.21	12.4	12	20 s/point-5/w-10	
			808 + 905	1,100	2	—	3.28	6.6	12	20 s/point-5/w-10	
Hsieh et al. (2012)	72	Laser	890	6,240	180	34.7	41.6	—	8	40 min-3/w-6	No beneficial effect in improving pain, physical activity, and postural stability is observed
Al Rashoud et al. (2014)	49	Laser	830	30	0.28		4	1.2	5	40 s/point-?-9	Short-term application of PBMT to specific acupuncture points in association with exercise or advice effectively relieves pain and improves quality of life
Alghadir et al. (2014)	40	Laser	850	50	Diameter = 1 mm	—	48	6	8	60 s/point-2/w-8	Effective in short-term pain relief and function improvement
Hinman et al. (2014)	282	—	—	10	—	—	—	0.2	6	20 min-1 or 2/w-8∼12	No significant improvements are observed
Kheshie et al. (2014)	53	Laser	830	800	—	25.6	50	1,250	—	1,953 s-2/w-12	Effective in improving pain and function
Helianthi et al. (2016)	62	Laser	785	50	—	25	2	4	5	80 s/point-2/w-10	Effective in reducing pain
Alfredo et al. (2018)	40	Laser	904	—	—	—	—	3	—	?-3/w-10	The immediate post-intervention improvements of PBMT plus strengthening exercises are maintained for 6 months
Braghin et al. (2019)	60	Laser	808	100	0.028	—	200	5.6	10	?-2/w-15	Combination of PBMT and exercise provides the best results for gait performance
Vassão et al. (2020a)	33	Laser	808	100	0.05	2000	91	4	14	40 s/point-2/w-16	PBMT does not optimize the effects of the exercise program
Vassão et al. (2020b)	62	Laser	808	100	0.05	2000	91	4	14	40 s/point-2/w-16	PBMT shows analgesic effects
Stausholm et al. (2022)	50	Laser	904	60	—	—	—	3	15	50 s/point-3/w-9	PBMT significantly reduces pain and provides a positive add-on effect in the follow-up period
Ahmad et al. (2023)	34	Laser	830	400	20	—	10–12	—	—	15 min-1/w-12	Incorporating HILT as an adjunct is more effective than PBMT in the improvements of pain, physical function and knee-related disability
Jankaew et al. (2023)	47	Laser	808/660	300	—	—	—	—	6	15 min-3/w-24	Significantly improves muscle strength and functional performance

One reason accounts for the controversial efficacy of PBMT in treating KOA patients might be the heterogeneity of the applied light parameters (Summarized in Table 1). According to the recommendation of World Association for Laser Therapy (WALT), light irradiation for the knee joint should obey the following doses: ≥4 J/point with 780–860 nm wavelength (mean power: 5–500 mW) and/or ≥1 J/point with 904 nm wavelength (mean power: 5–500 mW, peak power > 1,000 mW) respectively (Bjordal, 2012). Using meta-analysis, Stausholm et al. (2019) also gives the optimal range of wavelength and total energy that should be considered when applying PBMT. They elucidate that PBMT reduces KOA pain and disability at 4–8 J with 785–860 nm wavelength and at 1–3 J with 904 nm wavelength per treatment spot. In Malik’s recent study, PBMT reduces pain at 4–8 J with a wavelength of 640–905 nm per point applied twice a week for a total of 10–16 sessions (Malik et al., 2023). All of these researches provide reference for the dosage of applied PBMT. However, current studies have not reached a consistent conclusion on the light parameters that should be applied when treating KOA patients. In addition, the biphasic dose-response of PBMT, a phenomenon characterized by lower doses exerting stimulatory effects and higher doses display inhibitory or even detrimental effects (Chung et al., 2012; Nie et al., 2023), also needs to be taken into consideration when investigating the optimal light parameters. Apart from the light parameters displayed in Table 1, the irradiation location also matters. Feng et al. (2023) suggested the optimal irradiation location was on both sides of the patella, where the largest dose of PBMT could reach the articular cartilage.

## 3 Effects of PBMT in KOA tissue resilience and pain relief: preclinical evidence

### 3.1 PBMT ameliorates synovitis

KOA has been recognized as a whole joint disease, and synovitis is considered to be a promising precursor of KOA development instead of a secondary result of cartilage breakdown (Atukorala et al., 2016). According to Bacon’s study, a loss of 0.1 mm of cartilage thickness over 2 years was associated with an increase of 0.32 point in WOMAC pain in KOA patients, indicating a poor association between cartilage thickness loss and joint pain (Bacon et al., 2020). The exacerbation of pain was further demonstrated to be mediated by synovitis. This finding challenges the conventional chondroprotective therapies while treating KOA. In addition, synovitis is established to be strongly correlated with pain and disease severity of KOA (Ayral et al., 2005; Baker et al., 2010; Robinson et al., 2016). Therefore, anti-synovitis therapy might shed new light on developing novel effective interventions for KOA.

PBMT is capable of attenuating synovitis through several ways. First, PBMT is effective in modulating the expression of inflammatory factors (Alves et al., 2013; de Oliveira et al., 2017). Alves et al. (2013) evaluated the effects of an 808 nm PBMT with different output powers (50 mW vs. 100 mW) on the joint inflammation in papain-induced KOA rats. The results shown that PBMT with 50 mW was more efficient in modulating the expression of inflammatory mediators (IL-1β, IL-6) and the number of inflammatory cells (macrophages and neutrophils) and therefore significantly attenuated joint inflammatory process. When combined with exercise therapy, PBMT is also suggested to significantly increase the level of IL-10 (an anti-inflammatory factor) in KOA individuals (Vassão et al., 2021). Second, light irradiation contributes to macrophage repolarization in KOA synovium. Macrophages are highly plastic and can play dual modulatory roles in KOA synovitis because it can be polarized to different phenotypes, namely, the pro-inflammatory M1 phenotype and the anti-inflammatory/tissue-repairing M2 phenotype (Wynn and Vannella, 2016; Thomson and Hilkens, 2021). *In vitro* cell culturing experiments suggested that 440 s irradiation per day with the 810 nm PBMT (power density 2 mW/cm^2^, energy density 0.88 J/cm^2^) for 2 days can effectively inhibit the polarization of LPS and IFN-γ-induced bone marrow-derived macrophage (BMDM) to M1 phenotype (Li et al., 2020; Sun et al., 2020), providing a new idea for studying the possible mechanism of PBMT in alleviating KOA synovitis. Zhang’s previous work provides the first *in vivo* evidence for the effectiveness of PBMT in modulating synovial macrophage polarization in KOA mice (Zhang et al., 2022). A 630 nm Light Emitting Diode (LED) device was applied to treat collagenase-induced KOA mice with different power densities. Their results suggested that 630 nm LED-PBMT (power density 10 mW/cm^2^, 1 h per time, energy density 36 J/cm^2^) once a day for consecutive 4 weeks effectively alleviated KOA pain and synovitis in mice, and this amelioration might be partially due to the polarization of synovial macrophages from M1 to M2 phenotype according to the results of histomorphological analysis. Apart from these, PBMT can stimulate angiogenesis in synovium as well, which is one of the hallmarks of synovitis (Mapp and Walsh, 2012). da Rosa et al. (2012) analyzed the influence of PBMT at wavelengths of 660 and 808 nm in a papain-induced KOA rat model. Two weeks after the last injection of papain, KOA rats were treated with laser irradiation (660 nm or 808 nm, power density 3.57 W/cm^2^, 40 s per time) respectively. The energy used in both groups was 4 J. The results showed that both laser groups significantly increased new blood vessels formation and decrease fibrotic tissue formation in joint synovium tissue when compared to the control, which suggested PBMT stimulated angiogenesis and reduced fibrosis in KOA rats. And the stimulatory effects were demonstrated to be stronger in 808 nm PBMT group.

### 3.2 PBMT exerts chondroprotective effects

Extensive and irreversible degeneration of articular cartilage is the primary event in the onset and development of KOA. The regeneration of articular cartilage has long become a major topic of KOA treatment. In the context of KOA, the balance of anabolic and catabolic process of cartilage metabolism is broken, and inflammatory factors such as matrix metalloproteinases (MMPs) are released to facilitate cartilage degeneration (Yin et al., 2022). By applying PBMT, the production of catabolic factors, including IL-1β, inducible nitric oxide synthase (iNOS), tumor necrosis factor (TNF)-α, MMP-3, and MMP-13, is reduced, meanwhile the loss of anabolic factors, for example, collagen (Col) II and transforming growth factor (TGF)-β etc., slows down (Chung et al., 2012; Wang et al., 2014; Trevisan et al., 2020; Zhang et al., 2022). In KOA animals, PBMT has been demonstrated to be efficient in increasing other anabolic factors including IL-4, IL-10, proteoglycan and aggrecan, showing its role in maintaining tissue hemostasis and exerting chondroprotective effects (Balbinot et al., 2021; Tim et al., 2022). Lin et al. (2006) used He-Ne laser (632 nm, power density 3.1 mW/cm^2^, energy density 2.79 J/cm^2^) to treat papain-induced KOA rats for 15 min each time, three times per week for 8 weeks. Measured by histopathological staining, the density of mucopolysaccharide was found to significantly increase in treated rats than those of the controls, which was closely associated with the histopathological improvements, but conversely with the changes in KOA severity. These results indicate that PBMT is efficient in enhancing the biosynthesis of arthritic cartilage and in this way improve the histopathological changes in KOA.

PBMT displays positive roles in promoting chondrocytes proliferation and inhibiting their apoptosis. Using a 780 nm Ga-Al-As semiconductor laser to irradiate rabbit and human chondrocytes for 10 min per day for 5 days, Torricelli an colleagues found that no injury was observed in the cultured chondrocytes after irradiation and PBMT significantly stimulated chondrocyte proliferation and preserved the activity of chondrocytes 5 days after the irradiation (Torricelli et al., 2001). In Lin’s research, anterior cruciate ligament transection (ACLT) surgery was performed on the right knees of New Zealand white rabbits to establish the KOA rabbit model (Lin et al., 2012). Six weeks after surgery, rabbits in the PBMT group were given 810 nm laser irradiation (power density 5 mW/cm^2^, energy density 3 J/cm^2^) five times a week, 10 min each time for 2 weeks. After the ten-session laser illumination, the animals were sacrificed, and the results showed that PBMT could improve the cartilage structure, prevent articular cartilage degeneration, and significantly reduce the expression of caspase-3 in the surgery-induced KOA rabbits (Lin et al., 2012). In addition, PBMT is effective in recovering oxidative stress by modulating antioxidant enzymatic activity and thereby preserving the articular cartilage aspects (Martins et al., 2021).

However, not all studies support the beneficial effects of PBMT in protecting articular cartilage. Bayat et al. (2009) used 890 nm infrared laser (pulse mode, energy density 4.8 J/cm^2^) to treat rabbits with distal cartilage loss of right femoral patella groove twice a week. Through macroscopical and histological examination, the results of PBMT group were better than those of control group, but there was no significant difference between the two groups, indicating PBMT did not accelerate the healing of large osteochondral defects in rabbits. The reason for this result may be that PBMT is more likely to effectively promote cartilage repairing when the defect is minor than when the injury of cartilage is extensive or severe. This is consistent with the clinical experience because PBMT is suggested to be more effective in KOA patients at early stage of the disease instead of end stage.

### 3.3 PBMT attenuates KOA pain

Pain is the most common symptom in KOA patients, and is the primary reason for which patients seek medical consultations. Current conservative pharmacies remain relatively ineffective. Series of factors including damaged tissue, synovitis and central sensitization are shown to collectively contribute to OA-related pain (Raoof et al., 2018; Conaghan et al., 2019). PBMT can effectively lead to pain amelioration partially because its cardinal functions in resolving synovitis and reducing inflammatory factors releasing, but it can relieve pain through inhibiting central sensitization as well. In Yamada’s study, monosodium iodoacetate (MIA)-induced KOA rat model was used to test the effects of PBMT (904 nm, 6 or 18 J/cm^2^, 3 days per week, eight sessions in total) on oxidative stress and antioxidant capacity (Yamada et al., 2020). PBMT with 18 J/cm^2^ was demonstrated to be efficient in reducing pain and neutrophil activity in knee samples. Besides, PBMT was found to reduce oxidative stress damage at distant sites (blood serum and spinal cord), which may be a potential reason for pain relief because it reduced central sensitization. Balbinot et al. (2021) evaluated the efficacy of PBMT on cartilage degradation and spinal cord sensitization using MIA-induced Wistar rats. Their results supported the positive effects of PBMT in improving MIA-induced chronic pain-like behavior such as weight support and mechanical allodynia. Confirmed by immunohistochemistry, the effects of PBMT were associated to decreased contribution of spinal glial cells to pain-like behavior. Therefore, PBMT reduced the progression or maintenance of spinal cord sensitization, suggesting the potential role of PBMT in long-term central sensitization associated with chronic pain of KOA.

## 4 Conclusion and perspective

KOA is a highly prevalent disease all around the world bringing a huge socioeconomic burden. PBMT is efficient to treat KOA especially in the early stage of this disease. Despite the positive results achieved in both clinical and animal tests, many challenges still need to be addressed. For instance, the optimal light irradiation parameters (including wavelength, spot area, power density, energy density, frequency of application etc.) for treating KOA should be uncovered. To date, the most commonly used light source for PBMT is laser. However, LED also exerts similar therapeutic effects. Besides, LED may deliver much more energy because it has a broad irradiation area. And LED can be made into wearable devices for its property of flexibility, which might help chronic KOA patients to treat themselves at home. Besides, the underlying mechanisms of PBMT still need to be explored.

## References

[B1] Ahmad M. A. Hamid M. S. A. Yusof A. (2022). Effects of low-level and high-intensity laser therapy as adjunctive to rehabilitation exercise on pain, stiffness and function in knee osteoarthritis: a systematic review and meta-analysis. Physiotherapy 114, 85–95. 10.1016/j.physio.2021.03.011 34654554

[B2] Ahmad M. A. Moganan M. Hamid M. S. A. Sulaiman N. Moorthy U. Hasnan N. (2023). Comparison between low-level and high-intensity laser therapy as an adjunctive treatment for knee osteoarthritis: a randomized, double-blind clinical trial. Life (Basel) 13, 1519. 10.3390/life13071519 37511894 PMC10381799

[B3] Alfredo P. P. Bjordal J. M. Dreyer S. H. Meneses S. R. Zaguetti G. Ovanessian V. (2012). Efficacy of low level laser therapy associated with exercises in knee osteoarthritis: a randomized double-blind study. Clin. Rehabil. 26, 523–533. 10.1177/0269215511425962 22169831

[B4] Alfredo P. P. Bjordal J. M. Junior W. S. Lopes-Martins Rá B. Stausholm M. B. Casarotto R. A. (2018). Long-term results of a randomized, controlled, double-blind study of low-level laser therapy before exercises in knee osteoarthritis: laser and exercises in knee osteoarthritis. Clin. Rehabil. 32, 173–178. 10.1177/0269215517723162 28776408

[B5] Alghadir A. Omar M. T. Al-Askar A. B. Al-Muteri N. K. (2014). Effect of low-level laser therapy in patients with chronic knee osteoarthritis: a single-blinded randomized clinical study. Lasers Med. Sci. 29, 749–755. 10.1007/s10103-013-1393-3 23912778

[B6] Al Rashoud A. S. Abboud R. J. Wang W. Wigderowitz C. (2014). Efficacy of low-level laser therapy applied at acupuncture points in knee osteoarthritis: a randomised double-blind comparative trial. Physiotherapy 100, 242–248. 10.1016/j.physio.2013.09.007 24418801

[B7] Alves A. C. Vieira R. Leal-Junior E. dos Santos S. Ligeiro A. P. Albertini R. (2013). Effect of low-level laser therapy on the expression of inflammatory mediators and on neutrophils and macrophages in acute joint inflammation. Arthritis Res. Ther. 15, R116. 10.1186/ar4296 24028507 PMC3979014

[B8] Atukorala I. Kwoh C. K. Guermazi A. Roemer F. W. Boudreau R. M. Hannon M. J. (2016). Synovitis in knee osteoarthritis: a precursor of disease? Ann. Rheum. Dis. 75, 390–395. 10.1136/annrheumdis-2014-205894 25488799 PMC4916836

[B9] Ayral X. Pickering E. Woodworth T. Mackillop N. Dougados M. (2005). Synovitis: a potential predictive factor of structural progression of medial tibiofemoral knee osteoarthritis -- results of a 1 year longitudinal arthroscopic study in 422 patients. Osteoarthr. Cartil. 13, 361–367. 10.1016/j.joca.2005.01.005 15882559

[B10] Bacon K. LaValley M. Jafarzadeh S. Felson D. (2020). Does cartilage loss cause pain in osteoarthritis and if so, how much? Ann. Rheum. Dis. 79, 1105–1110. 10.1136/annrheumdis-2020-217363 32381567 PMC10406023

[B11] Baker K. Grainger A. Niu J. Clancy M. Guermazi A. Crema M. (2010). Relation of synovitis to knee pain using contrast-enhanced MRIs. Ann. Rheum. Dis. 69, 1779–1783. 10.1136/ard.2009.121426 20472593 PMC3885343

[B12] Balbinot G. Schuch C. P. Nascimento P. S. D. Lanferdini F. J. Casanova M. Baroni B. M. (2021). Photobiomodulation therapy partially restores cartilage integrity and reduces chronic pain behavior in a rat model of osteoarthritis: involvement of spinal glial modulation. Cartilage 13, 1309S–1321S. 10.1177/1947603519876338 31569995 PMC8804719

[B13] Bayat M. Kamali F. Dadpay M. (2009). Effect of low-level infrared laser therapy on large surgical osteochondral defect in rabbit: a histological study. Photomed. Laser Surg. 27, 25–30. 10.1089/pho.2008.2253 19196107

[B14] Beswick A. D. Wylde V. Gooberman-Hill R. Blom A. Dieppe P. (2012). What proportion of patients report long-term pain after total hip or knee replacement for osteoarthritis? A systematic review of prospective studies in unselected patients. BMJ open 2, e000435. 10.1136/bmjopen-2011-000435 PMC3289991 22357571

[B15] Bjordal J. M. (2012). Low level laser therapy (LLLT) and World Association for Laser Therapy (WALT) dosage recommendations. Photomed. Laser Surg. 30, 61–62. 10.1089/pho.2012.9893 22233559

[B16] Braghin R. Libardi E. C. Junqueira C. Rodrigues N. C. Nogueira-Barbosa M. H. Renno A. C. M. (2019). The effect of low-level laser therapy and physical exercise on pain, stiffness, function, and spatiotemporal gait variables in subjects with bilateral knee osteoarthritis: a blind randomized clinical trial. Disabil. Rehabil. 41, 3165–3172. 10.1080/09638288.2018.1493160 30324827

[B17] Bülow P. M. Jensen H. Danneskiold-Samsøe B. (1994). Low power Ga-Al-As laser treatment of painful osteoarthritis of the knee. A double-blind placebo-controlled study. Scand. J. Rehabil. Med. 26, 155–159. 10.2340/165019771994263155159 7801065

[B18] Cardoso F. D. S. Gonzalez-Lima F. Gomes da Silva S. (2021). Photobiomodulation for the aging brain. Ageing Res. Rev. 70, 101415. 10.1016/j.arr.2021.101415 34325071

[B19] Chow R. T. Johnson M. I. Lopes-Martins R. A. Bjordal J. M. (2009). Efficacy of low-level laser therapy in the management of neck pain: a systematic review and meta-analysis of randomised placebo or active-treatment controlled trials. Lancet 374, 1897–1908. 10.1016/s0140-6736(09)61522-1 19913903

[B20] Chung H. Dai T. Sharma S. K. Huang Y. Y. Carroll J. D. Hamblin M. R. (2012). The nuts and bolts of low-level laser (light) therapy. Ann. Biomed. Eng. 40, 516–533. 10.1007/s10439-011-0454-7 22045511 PMC3288797

[B21] Conaghan P. G. Cook A. D. Hamilton J. A. Tak P. P. (2019). Therapeutic options for targeting inflammatory osteoarthritis pain. Nat. Rev. Rheumatol. 15, 355–363. 10.1038/s41584-019-0221-y 31068673

[B22] da Costa B. R. Pereira T. V. Saadat P. Rudnicki M. Iskander S. M. Bodmer N. S. (2021). Effectiveness and safety of non-steroidal anti-inflammatory drugs and opioid treatment for knee and hip osteoarthritis: network meta-analysis. Bmj 375, n2321. 10.1136/bmj.n2321 34642179 PMC8506236

[B23] da Rosa A. S. dos Santos A. F. da Silva M. M. Facco G. G. Perreira D. M. Alves A. C. (2012). Effects of low-level laser therapy at wavelengths of 660 and 808 nm in experimental model of osteoarthritis. Photochem Photobiol. 88, 161–166. 10.1111/j.1751-1097.2011.01032.x 22053992

[B24] De Oliveira M. F. Johnson D. S. Demchak T. Tomazoni S. S. Leal-Junior E. C. (2022). Low-intensity LASER and LED (photobiomodulation therapy) for pain control of the most common musculoskeletal conditions. Eur. J. Phys. Rehabil. Med. 58, 282–289. 10.23736/s1973-9087.21.07236-1 34913330 PMC9980499

[B25] de Oliveira V. L. Silva J. A. Jr. Serra A. J. Pallotta R. C. da Silva E. A. de Farias Marques A. C. (2017). Photobiomodulation therapy in the modulation of inflammatory mediators and bradykinin receptors in an experimental model of acute osteoarthritis. Lasers Med. Sci. 32, 87–94. 10.1007/s10103-016-2089-2 27726041

[B26] Fang H. Beier F. (2014). Mouse models of osteoarthritis: modelling risk factors and assessing outcomes. Nat. Rev. Rheumatol. 10, 413–421. 10.1038/nrrheum.2014.46 24662645

[B27] Feng Z. Wang P. Song Y. Wang H. Jin Z. Xiong D. (2023). Photobiomodulation for knee osteoarthritis: a model-based dosimetry study. Biomed. Opt. Express 14, 1800–1817. 10.1364/boe.484865 37078045 PMC10110300

[B28] Fukuda V. O. Fukuda T. Y. Guimarães M. Shiwa S. de Lima Bdel C. Martins R. (2011). Short-term efficacy of low-level laser therapy in patients with knee osteoarthritis: a randomized placebo-controlled, double-blind clinical trial. Rev. Bras. Ortop. 46, 526–533. 10.1016/s2255-4971(15)30407-9 27027049 PMC4799277

[B29] GBD 2015 Disease and Injury Incidence and Prevalence Collaborators (2016). Global, regional, and national incidence, prevalence, and years lived with disability for 310 diseases and injuries, 1990-2015: a systematic analysis for the Global Burden of Disease Study 2015. Lancet 388, 1545–1602. 10.1016/s0140-6736(16)31678-6 27733282 PMC5055577

[B30] Gur A. Cosut A. Sarac A. J. Cevik R. Nas K. Uyar A. (2003). Efficacy of different therapy regimes of low-power laser in painful osteoarthritis of the knee: a double-blind and randomized-controlled trial. Lasers Surg. Med. 33, 330–338. 10.1002/lsm.10236 14677160

[B31] Gworys K. Gasztych J. Puzder A. Gworys P. Kujawa J. (2012). Influence of various laser therapy methods on knee joint pain and function in patients with knee osteoarthritis. Ortop. Traumatol. Rehabil. 14, 269–277. 10.5604/15093492.1002257 22764339

[B32] Hamblin M. R. (2013). Can osteoarthritis be treated with light? Arthritis Res. Ther. 15, 120. 10.1186/ar4354 24286607 PMC3978432

[B33] Hanna R. Dalvi S. Bensadoun R. J. Benedicenti S. (2021). Role of photobiomodulation therapy in modulating oxidative stress in temporomandibular disorders. A systematic review and meta-analysis of human randomised controlled trials. Antioxidants (Basel) 10, 1028. 10.3390/antiox10071028 34202292 PMC8300797

[B34] Helianthi D. R. Simadibrata C. Srilestari A. Wahyudi E. R. Hidayat R. (2016). Pain reduction after laser acupuncture treatment in geriatric patients with knee osteoarthritis: a randomized controlled trial. Acta Med. Indones. 48, 114–121. 27550880

[B35] Hinman R. S. McCrory P. Pirotta M. Relf I. Forbes A. Crossley K. M. (2014). Acupuncture for chronic knee pain: a randomized clinical trial. Jama 312, 1313–1322. 10.1001/jama.2014.12660 25268438

[B36] Hsieh R. L. Lo M. T. Liao W. C. Lee W. C. (2012). Short-term effects of 890-nanometer radiation on pain, physical activity, and postural stability in patients with knee osteoarthritis: a double-blind, randomized, placebo-controlled study. Arch. Phys. Med. Rehabil. 93, 757–764. 10.1016/j.apmr.2012.01.003 22459700

[B37] Huang Z. Chen J. Ma J. Shen B. Pei F. Kraus V. B. (2015). Effectiveness of low-level laser therapy in patients with knee osteoarthritis: a systematic review and meta-analysis. Osteoarthr. Cartil. 23, 1437–1444. 10.1016/j.joca.2015.04.005 PMC4814167 25914044

[B38] Jankaew A. You Y. L. Yang T. H. Chang Y. W. Lin C. F. (2023). The effects of low-level laser therapy on muscle strength and functional outcomes in individuals with knee osteoarthritis: a double-blinded randomized controlled trial. Sci. Rep. 13, 165. 10.1038/s41598-022-26553-9 36599881 PMC9812996

[B39] Karu T. (1999). Primary and secondary mechanisms of action of visible to near-IR radiation on cells. J. Photochem Photobiol. B 49, 1–17. 10.1016/s1011-1344(98)00219-x 10365442

[B40] Karu T. I. (2008). Mitochondrial signaling in mammalian cells activated by red and near-IR radiation. Photochem Photobiol. 84, 1091–1099. 10.1111/j.1751-1097.2008.00394.x 18651871

[B41] Katz J. N. Arant K. R. Loeser R. F. (2021). Diagnosis and treatment of hip and knee osteoarthritis: a review. Jama 325, 568–578. 10.1001/jama.2020.22171 33560326 PMC8225295

[B42] Kheshie A. R. Alayat M. S. Ali M. M. (2014). High-intensity versus low-level laser therapy in the treatment of patients with knee osteoarthritis: a randomized controlled trial. Lasers Med. Sci. 29, 1371–1376. 10.1007/s10103-014-1529-0 24487957

[B43] Khumaidi M. A. Paturusi I. Nusdwinuringtyas N. Islam A. A. Gunawan W. B. Nurkolis F. (2022). Is low-level laser therapy effective for patients with knee joint osteoarthritis? implications and strategies to promote laser therapy usage. Front. Bioeng. Biotechnol. 10, 1089035. 10.3389/fbioe.2022.1089035 36568305 PMC9773374

[B44] Kim B. Brandli A. Mitrofanis J. Stone J. Purushothuman S. Johnstone D. M. (2017). Remote tissue conditioning - an emerging approach for inducing body-wide protection against diseases of ageing. Ageing Res. Rev. 37, 69–78. 10.1016/j.arr.2017.05.005 28552720

[B45] Li K. Liang Z. Zhang J. Zuo X. Sun J. Zheng Q. (2020). Attenuation of the inflammatory response and polarization of macrophages by photobiomodulation. Lasers Med. Sci. 35, 1509–1518. 10.1007/s10103-019-02941-y 32065300

[B46] Lin H. He C. Luo Q. Zhang J. Zeng D. (2012). The effect of low-level laser to apoptosis of chondrocyte and caspases expression, including caspase-8 and caspase-3 in rabbit surgery-induced model of knee osteoarthritis. Rheumatol. Int. 32, 759–766. 10.1007/s00296-010-1629-5 21188382

[B47] Lin Y. Huang M. Chai C. (2006). Effects of helium-neon laser on the mucopolysaccharide induction in experimental osteoarthritic cartilage. Osteoarthr. Cartil. 14, 377–383. 10.1016/j.joca.2005.10.010 16359876

[B48] Malik S. Sharma S. Dutta N. Khurana D. Sharma R. Sharma S. (2023). Effect of low-level laser therapy plus exercise therapy on pain, range of motion, muscle strength, and function in knee osteoarthritis - a systematic review and meta-analysis. Somatosens. Mot. Res. 40, 8–24. 10.1080/08990220.2022.2157387 36576096

[B49] Mapp P. I. Walsh D. A. (2012). Mechanisms and targets of angiogenesis and nerve growth in osteoarthritis. Nat. Rev. Rheumatol. 8, 390–398. 10.1038/nrrheum.2012.80 22641138

[B50] Martins L. P. O. Santos F. F. D. Costa T. E. D. Lacerda A. C. R. Santos J. M. D. Costa K. B. (2021). Photobiomodulation therapy (Light-Emitting diode 630 nm) favored the oxidative stress and the preservation of articular cartilage in an induced knee osteoarthritis model. Photobiomodul Photomed. Laser Surg. 39, 272–279. 10.1089/photob.2020.4926 33497593

[B51] Nie F. Hao S. Ji Y. Zhang Y. Sun H. Will M. (2023). Biphasic dose response in the anti-inflammation experiment of PBM. Lasers Med. Sci. 38, 66. 10.1007/s10103-022-03664-3 36749428

[B52] Peplow P. V. Chung T. Y. Baxter G. D. (2012). Photodynamic modulation of wound healing: a review of human and animal studies. Photomed. Laser Surg. 30, 118–148. 10.1089/pho.2011.3142 22283621

[B53] Raoof R. Willemen H. Eijkelkamp N. (2018). Divergent roles of immune cells and their mediators in pain. Rheumatol. Oxf. 57, 429–440. 10.1093/rheumatology/kex308 PMC5850827 28968842

[B54] Rayegani S. M. Raeissadat S. A. Heidari S. Moradi-Joo M. (2017). Safety and effectiveness of low-level laser therapy in patients with knee osteoarthritis: a systematic review and meta-analysis. Lasers Med. Sci. 8, S12-S19–s19. 10.15171/jlms.2017.s3 PMC5642172 29071029

[B55] Robinson W. H. Lepus C. M. Wang Q. Raghu H. Mao R. Lindstrom T. M. (2016). Low-grade inflammation as a key mediator of the pathogenesis of osteoarthritis. Nat. Rev. Rheumatol. 12, 580–592. 10.1038/nrrheum.2016.136 27539668 PMC5500215

[B56] Sharma L. (2021). Osteoarthritis of the knee. N. Engl. J. Med. 384, 51–59. 10.1056/NEJMcp1903768 33406330

[B57] Soleimanpour H. Gahramani K. Taheri R. Golzari S. E. Safari S. Esfanjani R. M. (2014). The effect of low-level laser therapy on knee osteoarthritis: prospective, descriptive study. Lasers Med. Sci. 29, 1695–1700. 10.1007/s10103-014-1576-6 24733283

[B58] Stausholm M. Naterstad I. Joensen J. Lopes-Martins R. Sæbø H. Lund H. (2019). Efficacy of low-level laser therapy on pain and disability in knee osteoarthritis: systematic review and meta-analysis of randomised placebo-controlled trials. BMJ open 9, e031142. 10.1136/bmjopen-2019-031142 PMC6830679 31662383

[B59] Stausholm M. B. Naterstad I. F. Alfredo P. P. Couppé C. Fersum K. V. Leal-Junior E. C. P. (2022). Short- and long-term effectiveness of low-level laser therapy combined with strength training in knee osteoarthritis: a randomized placebo-controlled trial. J. Clin. Med. 11, 3446. 10.3390/jcm11123446 35743513 PMC9225274

[B60] Stelian J. Gil I. Habot B. Rosenthal M. Abramovici I. Kutok N. (1992). Improvement of pain and disability in elderly patients with degenerative osteoarthritis of the knee treated with narrow-band light therapy. J. Am. Geriatr. Soc. 40, 23–26. 10.1111/j.1532-5415.1992.tb01824.x 1727843

[B61] Sun J. Zhang J. Li K. Zheng Q. Song J. Liang Z. (2020). Photobiomodulation therapy inhibit the activation and secretory of astrocytes by altering macrophage polarization. Cell Mol. Neurobiol. 40, 141–152. 10.1007/s10571-019-00728-x 31446561 PMC11448964

[B62] Tang X. Wang S. Zhan S. Niu J. Tao K. Zhang Y. (2016). The prevalence of symptomatic knee osteoarthritis in China: results from the China Health and retirement longitudinal study. Arthritis Rheumatol. 68, 648–653. 10.1002/art.39465 26474054

[B63] Tascioglu F. Armagan O. Tabak Y. Corapci I. Oner C. (2004). Low power laser treatment in patients with knee osteoarthritis. Swiss Med. Wkly. 134, 254–258. 10.4414/smw.2004.10518 15243853

[B64] Thomson A. Hilkens C. M. U. (2021). Synovial macrophages in osteoarthritis: the key to understanding pathogenesis? Front. Immunol. 12, 678757. 10.3389/fimmu.2021.678757 34211470 PMC8239355

[B65] Tim C. R. Martignago C. C. S. Assis L. Neves L. M. Andrade A. L. Silva N. C. (2022). Effects of photobiomodulation therapy in chondrocyte response by *in vitro* experiments and experimental model of osteoarthritis in the knee of rats. Lasers Med. Sci. 37, 1677–1686. 10.1007/s10103-021-03417-8 34554354

[B66] Torricelli P. Giavaresi G. Fini M. Guzzardella G. A. Morrone G. Carpi A. (2001). Laser biostimulation of cartilage: *in vitro* evaluation. Biomed. Pharmacother. 55, 117–120. 10.1016/s0753-3322(00)00025-1 11293815

[B67] Trevisan E. S. Martignago C. C. S. Assis L. Tarocco J. C. Salman S. Dos Santos L. (2020). Effectiveness of led photobiomodulation therapy on treatment with knee osteoarthritis: a rat study. Am. J. Phys. Med. Rehabil. 99, 725–732. 10.1097/phm.0000000000001408 32167952

[B68] Vassão P. G. de Souza A. C. F. da Silveira Campos R. M. Garcia L. A. Tucci H. T. Renno A. C. M. (2021). Effects of photobiomodulation and a physical exercise program on the expression of inflammatory and cartilage degradation biomarkers and functional capacity in women with knee osteoarthritis: a randomized blinded study. Adv. Rheumatol. 61, 62. 10.1186/s42358-021-00220-5 34656170

[B69] Vassão P. G. de Souza M. C. Silva B. A. Junqueira R. G. de Camargo M. R. Dourado V. Z. (2020a). Photobiomodulation via a cluster device associated with a physical exercise program in the level of pain and muscle strength in middle-aged and older women with knee osteoarthritis: a randomized placebo-controlled trial. Lasers Med. Sci. 35, 139–148. 10.1007/s10103-019-02807-3 31144070

[B70] Vassão P. G. Silva B. A. de Souza M. C. Parisi J. R. de Camargo M. R. Renno A. C. M. (2020b). Level of pain, muscle strength and posture: effects of PBM on an exercise program in women with knee osteoarthritis - a randomized controlled trial. Lasers Med. Sci. 35, 1967–1974. 10.1007/s10103-020-02989-1 32157582

[B71] Wang P. Liu C. Yang X. Zhou Y. Wei X. Ji Q. (2014). Effects of low-level laser therapy on joint pain, synovitis, anabolic, and catabolic factors in a progressive osteoarthritis rabbit model. Lasers Med. Sci. 29, 1875–1885. 10.1007/s10103-014-1600-x 24890034

[B72] Wynn T. Vannella K. (2016). Macrophages in tissue repair, regeneration, and fibrosis. Immunity 44, 450–462. 10.1016/j.immuni.2016.02.015 26982353 PMC4794754

[B73] Yamada E. F. Bobinski F. Martins D. F. Palandi J. Folmer V. da Silva M. D. (2020). Photobiomodulation therapy in knee osteoarthritis reduces oxidative stress and inflammatory cytokines in rats. J. Biophot. 13, e201900204. 10.1002/jbio.201900204 31568634

[B74] Yin J. Zeng H. Fan K. Xie H. Shao Y. Lu Y. (2022). Pentraxin 3 regulated by miR-224-5p modulates macrophage reprogramming and exacerbates osteoarthritis associated synovitis by targeting CD32. Cell Death Dis. 13, 567. 10.1038/s41419-022-04962-y 35739102 PMC9226026

[B75] Yurtkuran M. Alp A. Konur S. Ozçakir S. Bingol U. (2007). Laser acupuncture in knee osteoarthritis: a double-blind, randomized controlled study. Photomed. Laser Surg. 25, 14–20. 10.1089/pho.2006.1093 17352632

[B76] Zhang R. Qu J. (2023). The mechanisms and efficacy of photobiomodulation therapy for arthritis: a comprehensive review. Int. J. Mol. Sci. 24, 14293. 10.3390/ijms241814293 37762594 PMC10531845

[B77] Zhang Y. Ji Q. Chen D. Chang B. Xu Y. Qiu H. (2022). Efficacy of 630 nm LED-based non-damage phototherapy on early stage knee osteoarthritis in mice. Chin. J. Laser Med. Surg. 31, 61–66+116-118. 10.13480/j.issn1003-9430.2022.0061

